# Selenium and Zinc Status in Chronic Myofascial Pain: Serum and Erythrocyte Concentrations and Food Intake

**DOI:** 10.1371/journal.pone.0164302

**Published:** 2016-10-18

**Authors:** João Araújo Barros-Neto, Adelmir Souza-Machado, Durval Campos Kraychete, Rosangela Passos de Jesus, Matheus Lopes Cortes, Michele dos Santos Lima, Mariana Carvalho Freitas, Tascya Morganna de Morais Santos, Gustavo Freitas de Sousa Viana, José Antonio Menezes-Filho

**Affiliations:** 1 College of Nutrition, Federal University of Alagoas, Maceió, Alagoas, Brazil; 2 Institute of Health Sciences, Federal University of Bahia, Salvador, Bahia, Brazil; 3 College of Medicine, Federal University of Bahia, Salvador, Bahia, Brazil; 4 College of Nutrition, Federal University of Bahia, Salvador, Bahia, Brazil; 5 Multidisciplinary institute in health, Federal University of Bahia, Vitória da Conquista, Bahia, Brazil; 6 University Center Estácio FIC, Department of Nutrition, Fortaleza, Ceará, Brazil; 7 Federal Institute of Alagoas, Maceió, Alagoas, Brazil; 8 College of Pharmacy, Federal University of Bahia, Salvador, Bahia, Brazil; University of South Alabama Mitchell Cancer Institute, UNITED STATES

## Abstract

**Introduction:**

Nutritional disorders have been reported to be important causal factors that can intensify or cause a painful response in individuals with chronic musculoskeletal pain.

**Aim:**

To assess the habitual intake of and the serum and erythrocyte levels of selenium and zinc in patients with chronic myofascial pain.

**Materials and Methods:**

A case-control study of 31 patients with chronic myofascial pain (group I) and 31 subjects without pain (group II). Dietary record in five days for assessing food intake were used. The serum and erythrocyte concentrations of selenium and zinc were analyzed using an atomic absorption spectrophotometry. Pain intensity was assessed using a visual analog scale.

**Results:**

The group of patients with chronic myofascial pain, compared with the control group, showed a lower erythrocyte concentration of selenium (79.46 ± 19.79 μg/L *vs*. 90.80 ± 23.12 μg/L; p = 0.041) and zinc (30.56 ± 7.74 μgZn/gHb *vs*. 38.48 ± 14.86 μgZn/gHb, respectively; p = 0.004). In this study, a compromised food intake of zinc was observed in the majority of the subjects in both groups. The selenium intake was considered to be safe in 80% of the subjects in both groups; however, the likelihood of inadequate intake of this mineral was twice as high in group I (49.5% *vs*. 24.4%, respectively). In the logistic regression analysis, the erythrocyte concentration of zinc was associated with the presence of pain. In each additional 1 mg of Zn^2+^ per gram of hemoglobin, a reduction of 12.5% was observed in the risk of the individual having chronic myofascial pain (B = -0.133; adjusted OR = 0.875, 95% CI = 0.803 to 0.954, Wald = 9.187, standard error = 0.044, p = 0.002). Physical inactivity and obesity were noted more commonly in group I compared with the control group.

**Conclusion:**

In this study, patients with chronic myofascial pain showed lower intracellular stores of zinc and selenium and inadequate food intake of these nutrients.

## Introduction

Myofascial pain syndrome (MPS) is defined as a regional pain that originates in a corresponding muscle area, and the referred pain is attributed to the presence of a “taut band” where one can locate the “myofascial trigger points” (MTrPs) [1][2]. However, the neurophysiology and pathogenesis of MPS and MTrP are controversial and have not been confirmed [3][4][5][6]. At present, the criteria established by Simons and Travell’s study seem to be the most accepted diagnostic method worldwide. These criteria increase the percentage of agreement between different researchers by considering the existence of MTrPs during muscle palpation with a typical pattern of referred pain as a manifestation of local response contractions or muscle contractions and the restricted joint mobility of the compromised muscles [6][7][8].

There are several mechanisms underlying the development of MTrPs and the appearance of MPS. Although there is no consensus diagnosis, the factors that may predispose individuals to the development of MTrPs are as follows: acute trauma, repeated micro trauma, lifestyle, posture, the lack of vitamins and trace elements, sleep disorders and joint problems that predispose to the development of micro traumas [9] or the maintenance of persistent and self-sustaining muscle contractions that produce tissue ischemia and metabolic impairment [2][10].

Relative ischemia, which is associated with muscle spasms, can cause significant damage to the affected tissues in MPS [11]. Evidence suggests increased inflammatory mediators and hypoxia in MPS [12], which may result in the synthesis and release of proinflammatory substances (tumor necrosis factor-alpha, histamine, kinin, substance P, interleukin-1, prostaglandin, leukotriene, somatostatin, and calcitonin gene-related peptide), which activate muscle nociceptors and promote oxidative stress and free radical formation in an acid environment [13]. This condition contributes to increased activity in the motor end plate, the onset of pain, hypersensitivity, allodynia and referred pain, which are all characteristics of active MTrPs [11,14].

The influence of antioxidants on painful nociception and the reduction of damage caused by free radicals in patients with chronic musculoskeletal pain are controversial [15][16][17][18]. A body of evidences suggests that deficiencies of selenium (Se) and zinc (Zn) participate in the pathophysiological process of painful musculoskeletal disease, which is associated with the presence of pain and other clinical manifestations [17][18][19][20][21]. This research aims to study the nutritional aspects of patients with chronic myofascial pain by assessing the habitual intake of and the serum and intracellular levels of selenium and zinc and correlating these measures to the clinical characteristics of these patients.

## Materials and Methods

### Ethics statement

This study was approved by the Ethics and Research Committee of the School of Nutrition of the Federal University of Bahia—number 03/08. The patients who were eligible to participate in the study signed an informed consent form before the participating in the study.

### Design of the study and sampling

This exploratory and observational case-control study consisted of adults with chronic myofascial pain who were followed in a specialized pain-care unit at a university hospital in northeastern Brazil.

To calculate the sample, we determined the mean values of serum concentrations and the standard deviations found for magnesium and calcium in the pilot project and the mean difference to be detected as significant based itself on original published studies on patients with chronic musculoskeletal pain. Considering a significance level of 5% and a power of 90%, we analyzed the expected sample size for each trace element.

The subjects of this study were divided into two groups: group I consisted of patients who were diagnosed with chronic myofascial pain, and group II consisted of healthy subjects, who were paired by age and socio-economic status.

### Criteria for inclusion and non-inclusion in the study

Group I (case) consisted of individuals of both genders who were diagnosed with chronic myofascial pain that was associated with the presence of MTrPs and muscle pain scores of > 4 assessed by the pain scale and who were aged 18–60 years. Group II (control) comprised family members and companions of patients who were from the same area or district, were of similar socioeconomic conditions and had undiagnosed pain.

Individuals were not included in this study if they were diagnosed with radiculopathy, degenerative or inflammatory joint disorder, central and peripheral neuropathies, nerve compression syndromes, multiple sclerosis, myasthenia gravis and polymyositis, systemic diseases such as hypothyroidism or severe metabolic or infectious diseases (herpes viruses, picornavirus, *Trichinella spiralis*, cysticercosis and toxoplasmosis, Hepatitis B, C, HTLV or HIV, chronic fatigue syndrome and fibromyalgia). Individuals with electrolyte disturbances or users of diuretics were also not included in this study.

#### Sociodemographic and socioeconomic indicators and lifestyle

Information regarding gender, age, marital status, and socioeconomic and environmental conditions was gathered.

For the assessment of lifestyle, information was collected on alcohol consumption, smoking and physical activity. Consumers of alcohol who reported drinking alcohol at least one or more times a month; former consumers or individuals with an intake interruption of >30 days before the start of the study and individuals who reported never having consumed alcohol were all considered. Smokers were classified as individuals who reported using tobacco in any frequency; former smokers were classified as individuals who had quit smoking at least one month prior to the study; and nonsmokers were classified as those who had never smoked. In the statistical analysis, former smokers were grouped with nonsmokers.

Individuals who reported the practice of moderate aerobic activity for at least 30 minutes/day, five days a week, or strenuous activity for at least 20 minutes/day, three days a week were considered to be physically active. Individuals who reported aerobic activity lower than the above-mentioned parameters were considered to be sedentary, according to the criteria of the American College of Sports Medicine and the American Heart Association [22].

### Nutritional parameters

#### Food survey

To assess the intake of selenium and zinc, dietary record in five days for assessing food intake were used. The average of the obtained results in dietary surveys collected was calculated using the following equation: Average intake = (Σ diet registry)/5.

To assess the intake of zinc and selenium, the calculated values in the food surveys were adjusted and corrected for the variability of consumption among individuals in the same group using the methodology described in the Dietary Reference Intakes (DRI) and the Estimated Average Requirement (EAR) and Recommended Dietary Allowance (RDA) as the cut-off points for estimating the requirements of these nutrients [23][24]. Whereas the distribution of zinc requirements tends to be asymmetric in the population, the evaluation of the intake of this nutrient using the EAR method may not be applicable. Thus, we adopted the classification proposed by the DRI (< EAR: inadequate; > EAR and < RDA: uncertain adequacy or inadequacy; > RDA: adequacy). To calculate the intake of micronutrients, we used the AVANUTRI^®^ software, version 3.09 (2008).

#### Measurements of weight, height and body mass index (BMI)

To obtain the weight measurement, a digital weight scale with a capacity of 200 kg and an accuracy of 50 g was used. Height was obtained using a portable stadiometer that was graduated in tenths of centimeters and affixed to a flat surface. The weight and height measurements were obtained and duplicated by different evaluators [25][26]. In the event of divergent values, a third measurement was performed, and the average of the three values was calculated. The height recorded was the mean of the two measurements. The BMI was obtained using the ratio between weight (kg) and squared height (m^2^). We considered the cut-off points of the WHO (1995) for eutrophic (18.5 kg/m^2^ and 24.9 kg/m^2^) and overweight (> 25 kg/m^2^) [27].

### Clinical evaluation of pain

The diagnosis of myofascial pain syndrome in any muscle was realized by specialized doctors after performing a thorough physical examination to identify the occurrence of MTrPs in one or more of the following muscles: the trapezius, infraspinatus, gluteus maximus, quadratus lumborum, and/or levator scapulae (cervical portion). The occurrence of MTrPs was diagnosed based on neck or shoulder pain that may or may not have been accompanied by the typical pattern of referred pain in the compromised muscle. After performing a thorough physical examination, MPS was diagnosed using the Simons Criteria, which required five major and at least one of the four minor criteria to be satisfied (**Major criteria**: 1—localized spontaneous pain; 2—spontaneous pain or altered sensations in the expected referred pain area for the given MTrP; 3—taut, palpable band in the accessible muscle; 4—exquisite, localized tenderness in a precise point along the taut band; and 5—some measurably reduced movement range. **Minor criteria**: 1—reproduction of spontaneously perceived pain and altered sensations by pressure on an MTrP; 2—elicitation of a local twitch response of the muscular fibers by transverse ‘snapping’ palpation or by needle insertion into the MTrP; 3—pain relief obtained by muscle stretching or dry needling injection of the MTrP; and 4—electromyographic demonstration of spontaneous electrical activity characteristic of active loci in the tender nodule of a taut band in the muscle) [7].

The pain intensity was evaluated using the VAS, which provides a simple and efficient measurement of the pain intensity and consists of a 10 cm horizontal line that measures the pain intensity reported by the patient at that moment. The pain intensity corresponds to an integer scale ranging from 0 to 10, where zero is an absence of pain and 10 is the worst pain possible [28]. The cutoff points established were as follows: 0, absence of pain; 1–3.9, mild pain; 4–7.9, moderate pain; and ≥ 8, intense pain.

### Blood collection

Blood was collected in two different vacuum tubes (Vacutainer^®^ BD). One tube with EDTA was used to analyze the trace minerals in erythrocytes and determine the hemoglobin (Hb) and cell counts using automated equipment. The other tube with no additive was used to determine the serum zinc and selenium measurements in participants who had previously fasted for at least 8 hours. The aliquots were centrifuged at 3,000 rpm for 15 minutes to obtain the erythrocyte mass and serum concentrations. After plasma removal, the erythrocyte mass was washed using 9% saline and centrifuged in triplicate at 10,000 rpm for 10 minutes. The serum and erythrocyte concentrations were fractioned and stored at -22°C until the analysis and determinations of selenium and zinc were performed.

### Determinations of total selenium (Se_T_) and Zn^2+^

The Zn^2+^ and Se_T_ concentrations and the serum and erythrocyte concentrations were determined using an atomic absorption spectroscopy in a graphite furnace, with Zeeman background correction, using a Spectra AA 240Z (Varian^®^) according to the conditions described in the manual. For the determination of zinc, the following conditions were used: wavelength (λ), 213.9 nm; gap, 0.7 nm; and flame, acetylene oxidant/air. Both measurements were performed for three readings with an integration time of 3 seconds.

For the determination of selenium, the red cell mass and serum samples were submitted to digestion with 2 mL of concentrated ultra-pure HNO_3_ (Merck^®^) and 500 μL of H_2_O_2_ on a heating plate at 50°C to 110°C for two hours. After the complete mineralization of the organic material, the solutions were transferred to a 5 mL volumetric flask, rinsed with type I pure water (Milli-Q, Millipore^®^) and subjected to a reading on the GTA 120 using palladium (Pd) and Mg(NO_3_)_2_ as a matrix modifier (200 μL of Pd and 125 μL of Mg(NO_3_)_2_ diluted in 400 μL of HNO_3_ 0.2%). The results are presented in μg/L. For quality control assurance in each batch of human serum, reference material from Seronorm^®^ Trace Elements Serum was analyzed. All of the samples were analyzed in duplicate.

The zinc serum concentration was obtained according to the method proposed by Rodriguez *et al*. [29], whereas the erythrocyte count was performed after the plasma separation and determined using the method described by Whitehouse *et al*. [30], with a standard curve diluted in a solution of 3% glycerol and 1% nitric acid. The zinc standard used was 1 g/L Titrisol^®^ (Merck). The Zn^2+^ concentrations were expressed in μgZn/gHb.

### Hemoglobin analysis in the red cell mass

To express the results, in terms of the mass of Zn/hemoglobin, an aliquot of 20 μL of erythrocyte lysate was used to determine the hemoglobin concentration, using the cyanmethemoglobin method from the Labtest Diagnostica kit [31].

### Statistics

The normal distribution of the variables was assessed using the Shapiro-Wilk test. The proportions were compared using the chi-square test or Fisher’s exact test. The magnitude of the association between X and Y was assessed using the odds ratio (OR). To analyze the differences in the means between both groups, we used Student’s *t* test and the nonparametric Mann-Whitney U test. The correlation between the continuous variables was analyzed using Pearson’s correlation test. A logistic regression model was proposed to investigate likely associations between the occurrence of pain and the explanatory (independent) variables. In these analyses, pain was assessed as a dependent variable, whereas the serum and erytrocyte concentrations and the food intake of selenium and zinc were assessed as an independent variable. Gender, the BMI, alcohol consumption and smoking were included in the analyses as potential sources of bias. P< 0.05 was considered significant in all tests. The analyses were performed using the Statistical software Package for Social Science (SPSS), version 17.0.

## Results

### General and anthroprometric characteristics

In both groups, the female subjects predominated (72.6%, n = 45); the highest frequency of women was observed in the group of patients with chronic pain (77.4% *vs*. 67.7%; Table 1). No differences between the groups in relation to the mean age (45.8 ± 9.14 years *vs*. 41.2 ± 9.50 years, p = 0.060), weight (71.4 ± 9.9 kg vs. 74.0 ± 16.1 kg, *p = 0*.*606*) or BMI (26.9 ± 3.9 kg/m^2^ *vs*. 25.1 ± 5.3 kg/m^2^, *p = 0*.*109*) were observed.

**Table 1:** Association of variables related to lifestyle and nutritional status in people with and without myofascial pain.

	Group I		Group II		
	*N*	*%*	*N*	*%*	*p*
**Gender**					
Men	07	22.6	10	32.3	0.393^†^
Women	24	77.4	21	67.7	
**Smoking**					
Yes	01	3.3	0	0	-
No	30	96.7	31	100.0	
**Alcoholism**					
Yes	04	12.9	13	41.9	0.021^♯^
No	27	87.1	18	58.1	
**Physical Activity**					
Yes	06	19.3	16	51.7	0.008^†^
No	25	80.7	15	48.3	
**BMI**					
Eutrophic	08	25.8	17	54.8	0.020^†^
Overweight	23	74.2	14	45.2	

Group I: Patients adults with chronic myofascial pain;

Group II: Individuals whitout chronic pain.

^†^ Pearson’s chi-square test;

^♯^ Fisher’s exact test; 95% CI (Confidence Interval);

BMI = Body mass index.

### Pain evaluation

Myofascial pain was classified as idiopathic or as having an unknown cause in 64.3% of the patients; 25% of the patients reported that the pain began after a work accident. Other causes, such as emotional origin or post-surgery, were reported in only 3.6% of patients for each cause. The intensity of pain indicated by the numerical pain scale showed a minimum value equal to five points and a maximum equal to 10 points, with a mean of 7.5 ± 1.4 points.

The presence of chronic myofascial pain was associated with physical inactivity (*p = 0*.*028*) and obesity (*p = 0*.*020*) (Table 1), but not with the intensity of referred pain (*p = 0*.*521*).

### Serum and erythrocyte concentrations of Se_T_ and Zn^2+^

**Fig 1:**
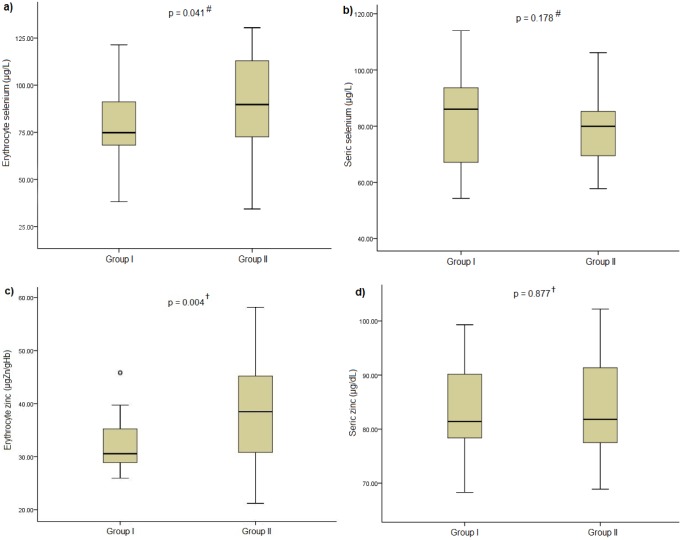
Erythrocyte and serum concentrations of Se_T_ and Zn^2+^ in adults with and without chronic myofascial pain. **a)** Erythrocyte concentration of Se_T_ in adults with chronic myofascial pain and without pain (μg/L); **b)** serum concentration of Se_T_ in adults with chronic myofascial pain and without pain (μg/L); **c)** erythrocyte concentration of Zn^2+^ in adults of both groups (μgZn/gHb); **d)** serum concentration of Zn^2+^ in adults of both groups (μg/dL). ^**#**^ Student´s t test; ^**†**^Mann-Whitney test.

Group I showed lower erythrocyte Se_T_ (79.46 ± 19.79 μg/L *vs*. 90.80 ± 23.12 μg/L) (*p = 0*.*041*) and Zn^2+^ (30.56 ± 7.74 μgZn/gHb *vs*. 38.48 ± 14.86 μgZn/gHb) (*p = 0*.*004*) compared with the control group (Fig 1).

The frequency of individuals with erythrocyte Zn^2+^ below the 1st quartile in this assessment was higher in the group of patients with chronic pain (93.6%, n = 29) than it was in the control group (51.6%, n = 16) (*p<0*.*001*) (Table 2). No differences between the groups were observed regarding the frequency of individuals with low serum levels of Se_T_ and Zn^2+^ (both *p>0*.*05*) (Table 2).

**Table 2:** Frequency of low concentrations of Zn^2+^ and Se_T_ in the serum and erythrocytes, and of inadequate intake of selenium and zinc.

	Group I		Group II		
	*n*	*%*	*n*	*%*	*P*
^a^**Erythrocyte Se**_**T**_					
1st quartile	13	41.9	03	9.7	0.008^#^
2nd, 3rd and 4th quartiles	18	58.1	28	90.3	
**Serum Se**_**T**_					
< 46 μg/L	01	3.2	01	3.2	1.000^#^
≥ 46 μg/L	30	96.8	30	96.8	
**Erythrocyte Zn**^**2+**^					
< 40 μgZn/gHb	29	93.6	15	48.4	0.000^#^
≥ 40 μgZn/gHb	02	6.4	16	51.6	
**Serum Zn**^**2+**^					
< 75 μg/dL	5	16.1	5	16.1	1.000^†^
≥ 75 μg/dL	26	83.9	26	83.9	
^b^ **Intake selenium**					
Z score ≤ -1	6	20.0	6	20.0	1.000^†^
Z score > -1	24	80.0	24	80.0	
^b^ **Intake zinc**					
< EAR	21	70.0	16	53.3	0.184^†^
≥ EAR	9	30.0	14	46.7	

Group I: Patients adults with chronic myofascial pain; Group II: Individuals whitout chronic pain.

^†^ Pearson’s chi-square test;

^#^Fisher’s exact test; 95% CI (Confidence Interval).

BMI = Body mass index;

EAR = estimated average requirement;

^a^1st quartile = 65.08μg Se/gHb;

^b^EAR as a reference.

### Dietary intake

We observed that higher proportion of patients with myofascial pain had inappropriately selenium intake compared to the control group.(49.5% *vs*. 24.4%, respectively). No significant difference in the average selenium intake between groups (45.17 ± 34.63 μg/day in group I *vs*. 56.88 ± 44.64 μg/day in group II) (*p = 0*.*261*) was observed. The group intake of zinc was assessed, and the median values for this nutrient were compared with the values established by the EAR. Males and females, in both groups presented a median intake of zinc that was lower than the EAR, and no difference in the median intake of this nutrient between the groups (*p = 0*.*162*) was observed.

In the individual assessment, 80.0% of the subjects in both groups showed a selenium intake, which was considered to be safe, whereas 70.0% (n = 21) of the subjects with chronic myofascial pain and 53.3% (n = 16) of the individuals in the control group had an inadequate zinc intake (Table 2).

### Selenium and zinc *vs*. chronic myofascial pain

The influence of selenium and zinc intake and concentrations of these minerals in serum and erythrocytes on the risk of chronic myofascial pain were analyzed using logistic regression. We showed an association between the concentration of Zn^2+^ in erythrocytes and pain, after the exclusion of serum and erythrocyte Se_T_, serum Zn^2+^ and selenium intake. Hence, in each additional 1 mg of Zn^2+^ per gram of hemoglobin, a reduction of 12.5% was observed in the risk of the individual having chronic myofascial pain (B = -0.133; adjusted OR = 0.875, 95% CI = 0.803 to 0.954, Wald = 9.187, standard error = 0.044, *p = 0*.*002*). When the increment of erythrocyte Zn^2+^ (5 μgZn/gHb) was calculated, there was a 48.6% reduction in the risk of myofascial pain (OR = 0.514).

## Discussion

In humans beings the concentrations of several trace elements with antioxidant activities have been extensively explored in the study of chronic myofascial pain. In the present study, the intracellular concentration (erythrocytes) of Se_T_ and Zn^2+^ showed a significant difference between the groups, and the concentration of Zn^2+^ in this compartment of the organism was associated with the presence of pain. The frequency of individuals with inadequate zinc intake was also higher among patients with chronic myofascial pain compared with subjects in the control group.

When assessing the concentrations of Zn^2+^ and Se_T_ in the serum and erythrocytes of the individuals in this study, we identified significant differences in the intracellular reserve, but not in the serum reserve, between the groups. The patients with chronic myofascial pain had lower erythrocyte selenium concentrations compared with those individuals without pain.

Evidence suggests that selenium and zinc status affects the humoral aspects of the immune function, which are linked to the inflammatory processes involving the production of reactive oxygen species (ROS) and redox control processes. The concentrations of these trace elements in erythrocytes constitute a parameter that is sensitive to the increased metabolic demand, which results from the chronic inflammatory processes observed in several pathologies [32][33][34][35] or severe nutritional deficiency [36][37]. Our findings are likely justified as a response to the oxidative stress that is commonly noted in chronic musculoskeletal pain and intensified by the inadequate intake of these micronutrients, particularly zinc, which is identified in this study population and is reflected in low intracellular concentrations of selenium and zinc.

Tissue damage found in patients with chronic pain induces the synthesis and release of inflammatory substances that increase the activity of the muscle motor endplate favoring the onset of pain [10][14]. Additionally, tissue damage induces oxidative stress by stimulating the production of free radicals by monocytes, macrophages and leukocytes, thereby increasing the demand for antioxidants [13].

Recent research on the trace elements involved in antioxidant activities in patients with chronic musculoskeletal pain was identified [16][17][18]. In these studies, as in our present study, low concentrations of Zn^2+^ are well documented.

Selenium deficiencies are still controversial [17][18][20][21]. The different results observed may be attributed to the various methods used for determining this element. Sendur *et al*. [17] found no differences in the serum selenium concentrations in patients with chronic musculoskeletal pain. However, the concentration of this trace element in serum does not represent the nutritional status of this nutrient. Such results suggest that selenium deficiency in these patients appears to be associated with a metabolic demand, which is chronic in nature, progressive and noticeable only in portions of the organism that reflect a deficiency in the organism’s reserves of this element, such as in erythrocytes.

To the best of our knowledge, this study is the first to quantitatively evaluate the dietary intake of zinc and selenium in patients with chronic myofascial pain. Group I was observed to have a higher percentage of inadequate intake of selenium and an increased frequency of individuals who exhibit a lower zinc intake compared with the estimated average requirement.

These results allow comparison with the usual consumption in the Brazilian population. Population-based studies in different Brazilian states have shown that the mean intake of zinc and selenium was 7.7 mg/day and 51.1 μg/day, respectively, and that the frequency of inadequate intake of these minerals also has values very close to those found in both groups of this study. The outcomes strongly suggest that within the Brazilian population, there is an inadequate eating habit, in which the ingestion of foods that are considered to be important sources of these nutrients does not appear to meet the EAR [38][39][40].

The values established as the EAR by the DRI do not sufficiently meet the metabolic demands imposed by oxidative stress on patients with chronic myofascial pain. Therefore, these EAR values are not applicable to this group of patients because the variables represent parameters only for healthy people.

Other important factors associated with the presence of chronic musculoskeletal pain and also observed in this study are related to the practice of physical activity and obesity. In this investigation, physical inactivity was observed more frequently in patients with chronic myofascial pain, which is a risk factor associated with painful symptoms, as reported in another study [41]. The practice of regular and guided physical activity has been suggested as an adjunctive treatment of pain because it activates muscle tension receptors during muscle contractions, thus causing the release of endogenous opioids, stimulating the release of endorphins from the pituitary gland and reducing the nociception of central and peripheral pain [42]. Physical activity, as an adjunctive pain treatment, also stimulates the growth of blood capillaries by increasing the oxygen supply, removing algogenic metabolic waste and reducing pain [43].

In the studied sample, myofascial pain was noted to be associated with obesity, which may be a risk factor for the development of pain or a consequence of the chronicity of the disease. Though the relationship between obesity and chronic musculoskeletal pain has not been elucidated, the frequency of obesity has been identified in 37% to 65% of patients with chronic musculoskeletal pain [44][45][46]. The association between obesity and chronic pain is most likely attributed to the increased mechanical load on weight-bearing joints. On the other hand, chronic myofascial pain leads to absenteeism from work and physical inactivity, thus becoming a risk factor for the development of obesity [10][46].

Additional investigations concerning the concentrations of these elements in other body compartments are warranted to better define the nutritional status of these minerals, which represent a major constraint for claiming the existence of deficiencies and their temporality (acute or chronic) in the present study. Another important limitation concerns the assessment of pain intensity. Although the visual analog scale is a valid and internationally accepted method for identifying the intensity of referred pain in patients with chronic musculoskeletal pain, this assessment is affected by the strong influences of other variables (such as emotional state, motivation for treatment, and drug therapy) and by its subjective method; hence, the results can be unreliable.

## Conclusion

In the present study, individuals with chronic myofascial pain exhibited low concentrations of Zn^2+^ and Se_T_. This group of patients also had a greater likelihood of inadequate intake of selenium and a higher frequency of individuals with an intake of zinc lower than the EAR. These findings are likely consequences of the inflammatory state and the need for mobilizing bodily reserves of these nutrients by an antioxidant demand.
